# Enhancing Horizontal Ratio of Transition Dipole Moment in Homoleptic Ir Complexes for High Outcoupling Efficiency of Organic Light‐Emitting Diodes

**DOI:** 10.1002/advs.202203903

**Published:** 2022-09-02

**Authors:** Jae‐Min Kim, Kyu Young Hwang, Sungmin Kim, Junseop Lim, Byungjoon Kang, Kum Hee Lee, Byoungki Choi, Seung‐Yeon Kwak, Jun Yeob Lee

**Affiliations:** ^1^ School of Chemical Engineering Sungkyunkwan University 2066, Seobu‐ro, Jangan‐gu Suwon Gyeonggi‐do 16419 Republic of Korea; ^2^ Samsung Advanced Institute of Technology Samsung Electronics Co. Ltd. Suwon Gyeonggi‐do 16678 Republic of Korea

**Keywords:** green organic light‐emitting diodes, iridium phosphorescent emitter, molecular orientation, outcoupling efficiency

## Abstract

The light‐emitting dipole orientation (EDO) of a phosphorescent emitter is a key to improving the external quantum efficiency (EQE) of organic light‐emitting diodes (OLEDs) without structural modification of the device. Here, four homoleptic Ir complexes as a phosphorescent emitter are systematically designed based on the molecular structure of tris(2‐phenylpyridine)iridium(III) (Ir(ppy)_3_) to control the EDO. Trimethylsilane, methyl, 2‐methylpropyl, and cyclopentylmethyl group substituted to pyridine ring of the ligand contribute to the improvement of the EDO from 76.5% for Ir(ppy)_3_ to 87.5%. A linear relationship between the EDO and the aspect ratio (geometric anisotropy factor) is founded, implying the importance of the effective area for the nonbonding force between host and dopant molecules. Also, it is investigated that the EDO enhancement mainly originates from the vertical alignment of the C3 axis of molecule in the substrate axis rather than the change in the direction of the transition dipole alignment in the molecular axis. The optical simulation reveals that the outcoupling efficiency of phosphorescent OLEDs adopting new dopants reaches 38.4%. The green OLEDs exhibiting 28.3% of EQE, 103.2 cd A^–1^ of current efficiency, and 98.2 lm W^–1^ of power efficiency are demonstrated, which is understood to have little electrical loss.

## Graphical abstract

Novel homoleptic Ir complexes exhibiting a high horizontal ratio of the transition dipole moment (EDO) are designed based on tris(2‐phenylpyridine)iridium(III). It is revealed that attachment of aliphatic moieties contributing to increase of geometric isotropic factor is a crucial strategy to enhance the EDO. A highly efficient phosphorescent organic light‐emitting diode with theoretical maximum external quantum efficiency is demonstrated.

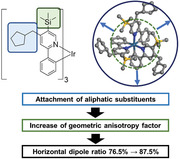

## 1 Introduction

For electronic devices, the portable display has been a critical component to intuitively communicate with the device, view multimedia contents, and obtain information from the internet. To reproduce real nature in the display, display technology has been developed toward high resolution, wide color coverage, high contrast ratio, and lightweight with low power consumption. After first commercialization, display adopting organic light‐emitting diodes (OLEDs) has been interested in consumers due to their superior performance and significantly developed in terms of efficiency, resolution, and operational stability. For the external quantum efficiency (EQE), the realization of phosphorescence pushed the efficiency beyond the limit of the exciton utilization efficiency of 25% in conventional fluorescence via the mechanism that harvests the triplet exciton of 75% in electrically generated excitons using heavy metal complexes.^[^ ^1^, ^2^ ^]^ In addition to the phosphorescence, thermally activated delayed fluorescence (TADF) and sensitized fluorescence (hyperfluorescence) were emerged as effective strategies to fully utilize singlet and triplet excitons with purely organic materials.^[^ ^3^ **^–^** ^6^ ^]^

After the invention of triplet harvesting mechanisms, highly efficient OLEDs reaching the theoretical maximum EQE calculated by classical dipole theory were reported.^[^ ^7^, ^8^, ^9^ ^]^ It was discussed that confinement of triplets, elimination of electrical loss, and good charge balance of exciplex‐forming cohost simultaneously contributed to realizing theoretical maximum EQE experimentally. Theoretical simulation indicated that enhancement of outcoupling efficiency would be a key for further increase of EQE since the internal quantum efficiency reached unity.^[^ ^10^ ^]^ Especially, among the approaches toward the increase of outcoupling efficiency, the increasing horizontal ratio of the transition dipole moment (TDM) of emitting dopant has been focused in many papers concerning materials design because it was the strategy enhancing the outcoupling efficiency without any external modification of the device.^[^ ^11^, ^12^, ^13^ ^]^ It was reported that the EQE of OLEDs could reach a value over 40% with a horizontal dipole ratio of 100% theoretically.^[^ ^12^ ^]^ With the roadmap toward ultimate EQE, the research focusing on the material design and elucidation of fundamental mechanisms underlying the preferred orientation have been widely performed in fluorescent, phosphorescent, and TADF emitters.^[^ ^14^, ^15^, ^16^, ^17^, ^18^, ^19^, ^20^, ^21^, ^22^, ^23^, ^24^, ^25^ ^]^ In the case of phosphorescent Ir complexes, most of the research addressed the orientation of heteroleptic Ir complexes rather than homoleptic structures. Comprehensive case studies via new materials design and physical investigations have suggested that the driving force toward the alignment of heteroleptic Ir complexes was derived by the geometrical and chemical origin. Since heteroleptic Ir complexes were configurated by a combination of main chromophore ligand and ancillary ligand, the geometrical shape of the molecule was similar to ellipsoid rather than sphere form.^[^ ^15^, ^26^ ^]^ This anisotropic geometry contributed to the increase of the possibility for horizontal orientation of the molecule. For the chemical view, the intermolecular force between emitting dopant and host molecules was investigated by using quantum chemical simulation and molecular dynamics modeling. It was reported that coulomb binding force and van der Waals force between dopant and host molecules predicted by the molecular dynamics modeling would be high enough to induce specific alignment during a vacuum deposition process.^[^ ^25^, ^26^, ^27^ ^]^ In the same context, it was discussed that the intrinsic nature of aromatic and aliphatic components in main and ancillary ligands was a critical point to rationalize the common phenomena for preferred alignment of heteroleptic Ir complexes.^[^ ^15^ ^]^ Meanwhile, a few studies investigating homoleptic Ir complexes were performed despite relatively better electrochemical stability. A recent paper discussed that geometric and chemical approaches were also effective in homoleptic structure as reported in the case of heteroleptic structure.^[^ ^28^ ^]^

In this work, we newly designed four homoleptic Ir complexes (GD1–4) showing preferred orientation via the addition of nonaromatic components based on the tris(2‐phenylpyridine)iridium(III) (Ir(ppy)_3_). The addition of trimethylsilane and cyclopentylmethyl to the pyridine part in the structure of Ir(ppy)_3_ (GD4) leads to the increase of the horizontal dipole ratio from 76.5% to 87.5%. The optical simulation revealed that the outcoupling efficiency of green phosphorescent OLEDs (PhOLEDs) was improved to 38.4% in the case of GD4 without any external device modification.

## 2 Results and Discussion

**Figure 1:**
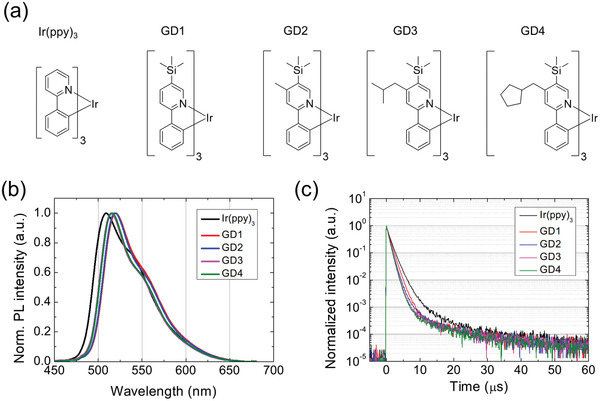
a) Molecular structures, b) PL spectra, and c) transient PL decay curves of GD1–4 with Ir(ppy)_3_ as reference material.

The molecular structure and photophysical properties of phosphorescent Ir complexes investigated in this work are described in **Figure** **1**. We selected Ir(ppy)_3_ as a reference homoleptic Ir complex and modified ligand structure to control the orientation of the TDM in the substrate axis. To minimize the electronic transition between ground and excited states resulting emission spectrum, aliphatic moieties, which were trimethylsilane, methyl, 2‐methylpropyl, and, cyclopentylmethyl were utilized to the modification of ligand structure. For common functional moiety, trimethylsilane was introduced to GD1–4 at the pyridine ring of the ligand. In addition to this modification, methyl (GD2), 2‐methylpropyl (GD3), and cyclopentylmethyl (GD4) were introduced to 4‐position of pyridine ring to accelerate the effect toward horizontal dipole orientation. Detailed information for materials synthesis is presented in supporting information.

Figure 1b shows photoluminescence (PL) spectra of newly designed homoleptic Ir phosphorescent dopants in a film state. PL spectra in the solution state are presented in Figure S1 in the Supporting Information. The peak wavelengths of the spectra were 520, 519, 515, and 516 nm for GD1–4, respectively. The shift of the emission spectra between GD4 and Ir(ppy)_3_ was 7 nm. The full width at half the maximum of the spectra was reduced from 63 to 57 nm. To investigate the radiative decay of exciton, transient PL characteristics were measured and presented in Figure 1c. The exciton lifetimes determined by the exponential fitting were 1.55/1.74/1.68/1.58 µs for GD1–4, respectively, which was shorter than that (1.95 µs) of Ir(ppy)_3_. Considering overall photophysical characteristics, it could be deduced that the electronic processes about the excitation and relaxation were not significantly affected by the addition of functional moieties. The distributions of the highest occupied molecular orbital (HOMO) and lowest unoccupied molecular orbital (LUMO) were calculated by using a quantum chemical calculation to verify the effect of the substitution. (Figure S2, Supporting Information) The information of the quantum chemical simulation is described in the Experimental Section. For all homoleptic dopants, HOMO was mainly distributed around Ir atom and partly located at the benzene ring of the ligand. Also, LUMO was mainly delocalized at the pyridine part. The distributions of HOMO and LUMO were not expanded to trimethylsilane, methyl, 2‐methylpropyl, and cyclopentylmethyl in GD1–4, supporting the little contribution of functional addition to the electronic transition. The transition between metal and ligand could be also deduced by the calculation results. Experimental HOMO and LUMO levels determined from the cyclic voltammetry and bandgap energy were −5.45/−2.97, −5.45/−2.97, −5.38/−2.90, −5.39/−2.91, and −5.39/−2.91 eV for Ir(ppy)_3_ and GD1–4, respectively. The cyclic voltammetry data are presented in Figure S3 in the Supporting Information.

**Figure 2:**
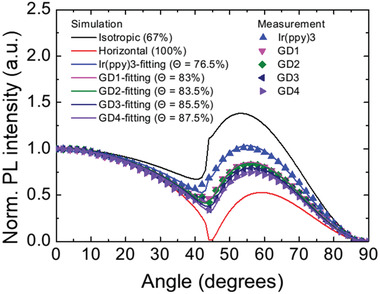
Angle‐dependent p‐polarized PL intensity of Ir complex‐doped film with optical simulation. The wavelength for dipole orientation analysis was determined by the wavelength corresponding to the maximum PL intensity.

The orientation of the transition dipole moment was analyzed by measuring angle‐dependent PL spectra of films.^[^ ^29^ ^]^ 2‐phenyl‐4,6‐bis(12‐phenylindolo[2,3‐a] carbazole‐11‐yl)‐1,3,5‐triazine was used as a host material and the doping concentration of Ir complexes was 3 wt%.^[^ ^30^ ^]^ **Figure** **2** shows p‐polarized PL intensities depending on the detection angle with optical simulation considering the ratio between horizontal and vertical components of the emitting dipoles. The wavelength used for the orientation analysis was determined as a wavelength corresponding to maximum intensity. The experimental results were fitted by the optical simulation very well. The horizontal ratios of the TDM were determined to 83%/83.5%/85.5%/87.5% for GD1–4, respectively. Ir(ppy)_3_ showed the horizontal dipole ratio of 76.5%, indicating preferred orientation rather than isotropic alignment (67%). The results exhibited that the attachment of additional moieties effectively contributed to enhancing the horizontal dipole ratio.

To understand the mechanism underlying induced alignment of Ir complexes in host matrix, ground, and excited states related to nonbonding forces were investigated by using quantum chemical simulation. Theoretically, two conditions should be simultaneously fulfilled for the preferred orientation of emitting dopant. First, the TDM should be anisotropic orientation. For homoleptic Ir complexes, the electronic transition during excitation occurred as a charge transfer process between metal and three ligands. Therefore, the spatial relationship between three vectors for TDM should not be orthogonal to each other for the net orientation of the moment vectors. Second, the orientation of the molecule should show anisotropic characters in the space of substrate coordinates, indicating the actual orientation of dopants induced by intermolecular forces. The TDM vectors for triplet states were calculated and visualized with optimized geometry in Figure S4 in the Supporting Information. All Ir complexes commonly showed net orientation of TDM between Ir atom and center of one ligand among three main ligands. Specifically, the angles between TDM vector and C3 axis for GD1–4 were 89.7°/89.5°/87.2°/86.7°, respectively. Ir(ppy)_3_ showed an angle of 85.1°. All Ir complexes exhibited an almost orthogonal relationship with the C3 axis.

**Figure 3:**
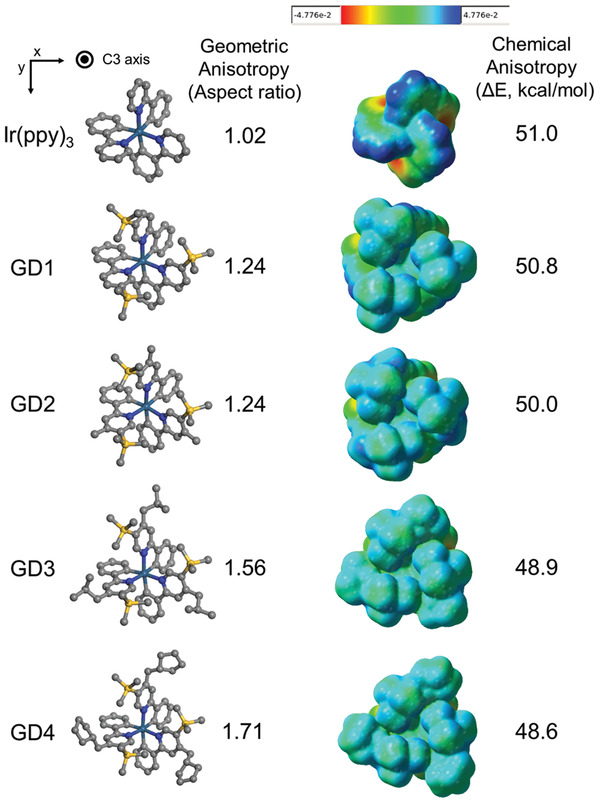
Optimized geometry and electrochemical isosurface of homoleptic Ir complexes. The geometric and chemical anisotropy factors were derived by the aspect ratio and the difference between maximum and minimum values in the electrochemical potential range. (isovalue = 0.001 electrons bohr^−3^)

The optimized geometries and the electrochemical potential isosurfaces of Ir complexes for the top view along the C3 axis are presented with anisotropy factors in **Figure** **3**. To correlate the orientation of dopants with molecular characteristics, geometric and chemical anisotropy were also calculated and depicted with molecular structures.^[^ ^21^, ^24^, ^31^ ^]^ For geometric anisotropy, the aspect ratio was derived by the ratio between long diameter (R) and short diameter (r) assuming that 3‐dimensional shape of the homoleptic Ir complexes was geometrically treated as a truncated cone as depicted in Figure S5 in the Supporting Information. In the side view, the long and short diameters were dependent on the substituents attached at the pyridine and benzene rings, respectively. Since the ligand modification in GD1–4 was performed at the pyridine part of the ligands, the increase of the aspect ratio originated from the increase of the long diameter (*R*) rather than the short diameter (*r*). In the case of chemical anisotropy, the difference between maximum and minimum values in electrochemical potential isosurface (with density isovalue = 0.001 electrons bohr^−3^) was determined as a representative value. The aspect ratios for Ir(ppy)_3_ and GD1–4 were 1.02/1.24/1.24/1.56/1.71, respectively. In view along the C3 axis, the distance between Ir atom and outermost atoms was increased by substituting the trimethylsilane moiety at the pyridine ring as the common modification of GD1–4 compared to Ir(ppy)_3_. Additional attachment of methyl at four‐position in GD2 could not significantly contribute to enhancing the geometrical anisotropy since the addition could not further increase the diameter in *x*–*y* space. However, the substituent elongation using 2‐methylpropyl and cyclopentylmethyl moiety in GD3 and GD4 effectively led to expanding the dimension in *x*–*y* space due to the effective length of the block. Specifically, the C–C chain between the outmost substituent and pyridine moiety mainly contributed to the increase of the aspect ratios compared to GD1 and GD2. In contrast to the geometric consideration, a variety of electrochemical potential did not show a meaningful difference in homoleptic Ir complexes. It was attributed that the nature of electron‐donating or withdrawing in substituents of GD1–4 was not strong due to the aliphatic structure consisting of carbon and silicon atoms only. Considering the trend of both anisotropy factors with the horizontal dipole ratio of Ir complexes, it could be deduced that the geometrical shape was mainly involved in the mechanism governing preferred orientation rather than coulomb forces by the electrochemical potential in this study.

**Figure 4:**
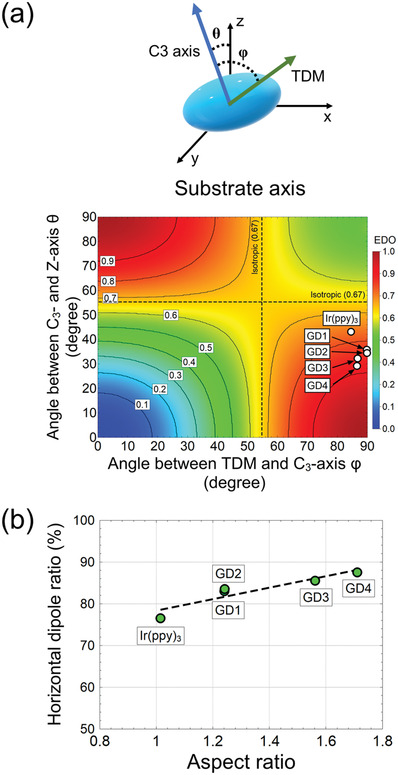
Correlation between molecular characteristics and the horizontal dipole ratio. a) Quantitative dependency of the horizontal dipole ratio on the spatial orientation of the molecules and TDM in the space of substrate axis. Since the angle between TDM and C3 axis for heteroleptic Ir complexes was close to the orthogonal relationship, the enhancement of the horizontal dipole ratio originated from the orientation of the C3 axis toward the synchronization with the *Z*‐axis in substrate space. b) The experimental horizontal dipole ratio according to the geometric anisotropy factor (aspect ratio).

**Figure** **4**a shows the predicted horizontal dipole ratio according to the relationship between TDM vector and C3 axis in substrate axis for better understanding of preferred orientation.^[^ ^21^ ^]^ To enhance the outcoupling efficiency of OLEDs, the orientation of the TDM vector in emitting dopant should be aligned along horizontal direction in molecular axis with absolute horizontal orientation in substrate axis. Therefore, the horizontal dipole ratio was dependent on the angle between the TDM and C3 axis (*φ*) and the angle between the C3 axis and *Z*‐axis of substrate space (*θ*). Since the TDMs of GD1–4 were almost orthogonal to the C3 axis in the molecular axis, the enhancement of horizontal dipole ratio due to the modification of ligand originated from the actual horizontal alignment of molecules rather than the change of direction in the TDM vector. In other words, the decrease of the angle between the C3‐ and *Z*‐axis from 43° for Ir(ppy)_3_ to 29° for GD4 mainly contributed to the accelerate horizontal dipole ratio. The physical parameters of the phosphorescent emitters were summarized in **Table** **1**.

**Table 1:** Photophysical and orientation (EDO)‐related parameters of homoleptic Ir complexes

	*λ*_PL_ ^a)^ [nm]	FWHM [nm]	*τ* [µs]	*E*_T1_ ^b)^ [eV]	PLQY [%]	*φ* ^c)^ [degree]	*θ* ^d)^ [degree]	EDO [%]
Ir(ppy)_3_	509	63	1.95	2.57	73.2	85.1	43	76.5
GD1	520	59	1.55	2.51	81.7	89.7	36	83.0
GD2	519	58	1.74	2.53	70.5	89.5	34	83.5
GD3	515	57	1.68	2.54	70.9	87.2	32	85.5
GD4	516	57	1.58	2.54	73.5	86.7	29	87.5

^a)^ The wavelength corresponding to the maximum intensity;

^b)^ The value at the onset of the PL spectrum;

^c)^ The angle between the TDM vector and C3 axis;

^d)^ The angle between the C3‐ and *Z*‐axis.

The effect of the geometric anisotropy factor on the horizontal dipole ratio is displayed in Figure 4b. As reported in the previous paper, Ir complexes investigated in this work showed a linear relationship between the horizontal dipole ratio and geometric anisotropy factor. The clear relationship might originate from the fact that GD1–4 were designed by the modification of ligand based on the same reference structure.

**Figure 5:**
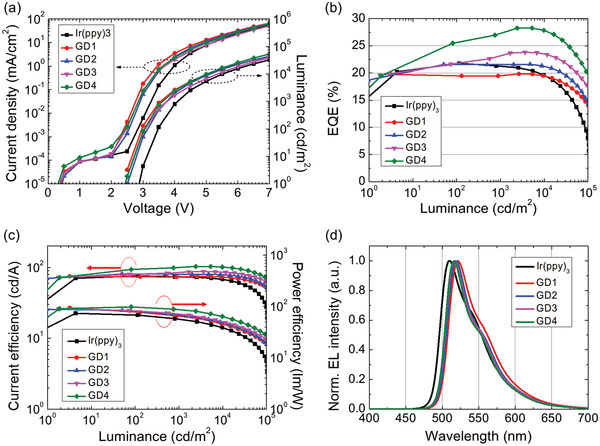
a) Current density‐voltage‐luminance, b) EQE‐luminance, c) current efficiency‐luminance‐power efficiency, and d) electroluminescence spectra of PhOLEDs adopting heteroleptic Ir complexes as an emitting dopant.

Green PhOLEDs adopting homoleptic Ir complexes were fabricated and device performance is depicted in **Figure** **5**. The device structure and molecular structure of organic materials used in PhOLEDs are presented in Figure S6 in the Supporting Information. The driving voltages corresponding to the luminance of 3000 cd m^–2^ were 4.1/4.2/4.3/4.1 V for GD1–4, which were lower than that of Ir(ppy)_3_ device (4.6 V) (Figure 5a). To understand the common reduction of driving voltage in the GD1–4 devices, the single carrier devices were fabricated to investigate the charge transport in the emitting layer. The device structures of single carrier devices are described in the Experimental Section. The current density (*J*)–voltage (*V*) characteristics of the single carrier devices are presented in Figure S7a,b in the Supporting Information. To correlate the *J*–*V* result with the driving voltages of PhOLEDs, we quantitatively analyzed the change of current densities in the GD1–4 single carrier devices along with that of Ir(ppy)_3_ device at 6 V as described in Figure S7c in the Supporting Information. For all single carrier devices adopting newly designed Ir complexes, the absolute values of the current density increase in the electron‐only devices were significantly higher than those for the current density decrease of the hole‐only devices. Considering the analysis, it could be deduced that the reduced driving voltages of GD1–4 devices relative to the Ir(ppy)_3_ device resulted from the enhancement of electron transport in the emitting layer. A similar range of the current densities in GD1–4 devices indicated that the electronic characteristics of dopants were not significantly changed due to the aliphatic substituents. Figure 5b depicts the EQE of PhOLEDs depending on the luminance. The EQEs of green PhOLEDs were 21.8%/19.8%/21.6%/23.8%/28.3% for Ir(ppy)_3_ and GD1–4 devices. The EQE of PhOLEDs increased from 21.8% for Ir(ppy)_3_ device to 28.3% for GD4 device.

Current and power efficiencies of PhOLEDs are depicted in Figure 5c. Maximum current efficiencies were 75.7/74.9/80.7/87.1/103.2 cd m^–2^ for Ir(ppy)_3_ and GD1–4 devices. Relatively low roll‐off characteristics of GD1–4 devices compared to that of Ir(ppy)_3_ would result from a shorter exciton lifetime of GD1–4. (Figure 1c) Electroluminescence spectra of PhOLEDs are displayed in Figure 5d. As expected from the PL spectra, the spectrum shift induced by the ligand modification was small as 7–13 nm. As a result, the Commission Internationale de l'Eclairage (CIE) color coordinates were slightly changed from (0.27, 0.64) for Ir(ppy)_3_ to (0.28, 0.65) for GD4. The device performance of PhOLEDs is summarized in **Table** **2**.

**Table 2:** Device performance of PhOLEDs adopting Ir complexes as a phosphorescent emitter

	Voltage [V]	EQE [%]		PE [lm W^–1^]		CE [cd A^–1^]		CIE coordinates	
	[3000 cd m^–2^]	Max.	[3000 cd m^–2^]	Max.	[3000 cd m^–2^]	Max.	[3000 cd m^–2^]	*x*	*y*
Ir(ppy)_3_	4.6	21.8	20.9	73.7	49.8	75.7	72.3	0.27	0.64
GD1	4.1	19.8	19.8	94.1	57.8	74.9	74.8	0.31	0.65
GD2	4.2	21.6	21.6	88.2	60.4	80.7	80.1	0.30	0.65
GD3	4.3	23.8	23.8	91.0	63.1	87.1	87.0	0.29	0.65
GD4	4.1	28.3	28.3	98.2	79.3	103.3	103.2	0.28	0.65

**Figure 6:**
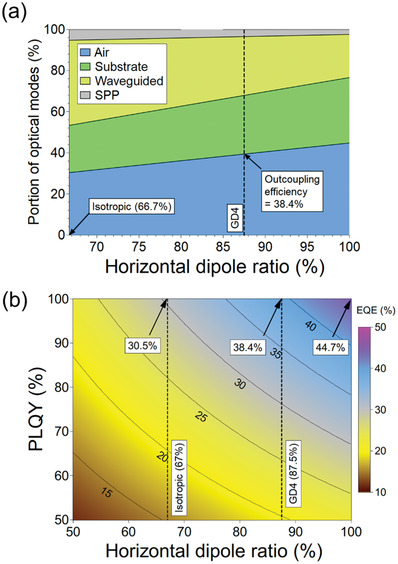
a) Optical modes analysis for various horizontal dipole ratios in PhOLEDs. PL spectrum of GD4 was used as a source spectrum. b) Theoretical maximum EQE depending on PLQY of the emitting layer and the horizontal dipole ratio of dopant. The charge balance factor was assumed as unity.

An optical simulation based on the classical dipole theory was performed to understand the EQE and outcoupling loss in the device structure.^[^ ^32^, ^33^, ^34^, ^35^, ^36^ ^]^ **Figure** **6** shows the optical modes depending on the horizontal dipole ratio and EQE simulation as a function of the photoluminescence quantum yield (PLQY) and the horizontal dipole ratio. Refractive indexes of organic materials were measured by using ellipsometry.

Under the assumption that the PL spectrum was equal to that of GD4, the portion of optical modes was calculated in the case of various horizontal dipole ratios (Figure 6a). As the horizontal dipole ratio increases, the optical mode corresponding to the outside of the device (air) was enhanced from 30.5% for isotropic orientation to 38.4% for GD4 due to the reduction of waveguided and substrate modes. Considering that the PLQY of GD4 in 3 wt%‐doped film was 74%, the theoretical maximum EQE of PhOLEDs adopting GD4 was calculated to 28.4% in the case of device structure fabricated in this work, which was close to the experimental value of 28.3%. It indicated that the charge balance factor was close to unity in the GD4 device. Figure 6b shows the contour plot of the EQE according to the PLQY and the horizontal dipole ratio. If the horizontal dipole ratio was assumed as 67% (isotropic), the maximum EQE would be limited to 30.5% even if the PLQY was 100%. However, the maximum EQE could be further enhanced to 44.7% in the case that the alignment of TDM was perfectly horizontal. This result implied that EQE over 40% could be realized by elaborate ligand design of Ir complexes without any external modification of geometric device architecture.

## 3 Conclusion

In summary, the enhancement of the horizontal dipole ratio of the homoleptic Ir complex was facilitated by the substitution of aliphatic functional moieties based on Ir(ppy)_3_ structure as a reference. To enhance the preferred orientation of Ir complexes, trimethylsilane, methyl, 2‐methylpropyl, and cyclopentylmethyl were introduced to the pyridine ring of Ir(ppy)_3_ structure. As a result, the horizontal dipole ratio of the homoleptic Ir complex was enhanced from 67.0% for Ir(ppy)_3_ to 87.5% for GD4 with minimization of the shift of emission spectrum. Green PhOLED adopting GD4 as a phosphorescent emitter exhibited the EQE of 28.3% with a CIE coordinate of (0.28, 0.65), which was close to the theoretical maximum value considering the PLQY and horizontal dipole ratio. The design approach to control the horizontal dipole ratio of homoleptic Ir complexes in this work would be applied to other homoleptic Ir complexes as a way of EQE enhancement of PhOLEDs.

## 4 Experimental Section

Detailed information on the synthesis of Ir complexes is presented in the Supporting Information. PL spectra were measured by using a fluorescence spectrophotometer (PerkinElmer, Waltham, MA; LS55). Device performance was characterized through Keithley 2400 measurement system and CS 2000 (Konica Minolta Inc.) spectroradiometer. PLQY and transient PL were measured by Hamamatsu Quantaurus‐QY C11347‐11 spectrometer and Quantaurus‐Tau fluorescence lifetime measurement system (C11367‐31, Hamamatsu Photonics). Angle‐dependent PL measurements to determine the EDO were carried out using a combination of a half‐cylinder lens on rotation stage (McScience), He‐Cd laser (Melles Griot Co.), and Maya 2000 Pro (Ocean Optics Inc.). Cyclic voltammetry was performed by using glassy carbon, platinum wire, and Ag/AgCl solution as working, counter, and reference electrodes. Tetrabutylammonium perchlorate was used as an electrolyte.

The PhOLEDs were fabricated by the vacuum deposition process. Organic layers and electrode were deposited with 1 Å s^–1^ on a wet‐cleaned indium tin oxide substrate. After the deposition, the devices were encapsulated with a moisture getter in the glovebox.

Geometry of the ground and excited states were optimized with the B3LYP exchange‐correlation functional, where the double‐zeta quality LANL2DZ basis set for Iridium atom and the 6‐31G** basis set for all other atoms.^[^ ^37^, ^38^ ^]^ An effective core potential was used to describe the inner core electrons of the Ir atom. The frontier molecular orbital analysis and electrostatic potential calculation of the optimized geometries were also calculated at the same level. Single point time‐dependent density functional theory (TD‐DFT) calculations under the Tamm‐Dancoff approximation for first excited triplet states were performed to compute the TDM for phosphorescent emission.^[^ ^39^ ^]^ During TD‐DFT calculation, nonperturbative two‐component zero‐order regular approximation was considered to treat the relativistic effect efficiently.^[^ ^40^ ^]^ The calculations were done using the Jaguar software package on the Schrodinger Material Science Suite.^[^ ^41^, ^42^ ^]^

## Conflict of Interest

The authors declare no conflict of interest.

## Supporting information

Supporting Information

## Data Availability

Research data are not shared.
