# Supplementary material for: Enhancing Horizontal Ratio of Transition Dipole Moment in Homoleptic Ir Complexes for High Outcoupling Efficiency of Organic Light‐Emitting Diodes
Source: Adv Sci (Weinh). 2022 Sep 2;9(31):2203903. doi: 10.1002/advs.202203903 (PMC9631091; doi:10.1002/advs.202203903)

ADVANCED SCIENCE
Open Access

Supporting Information

for *Adv. Sci.*, DOI 10.1002/advs.202203903

Enhancing Horizontal Ratio of Transition Dipole Moment in Homoleptic Ir Complexes for High Outcoupling Efficiency of Organic Light-Emitting Diodes

Jae-Min Kim, Kyu Young Hwang, Sungmin Kim, Junseop Lim, Byungjoon Kang, Kum Hee Lee, Byoungki Choi, Seung-Yeon Kwak and Jun Yeob Lee**

Supporting Information

Enhancing horizontal ratio of transition dipole moment in homoleptic Ir complexes for high outcoupling efficiency of organic light-emitting diodes

Jae-Min Kim[1,+], Kyu Young Hwang[2,+], Sungmin Kim[2,+], Junseop Lim[1+], Byungjoon Kang[2], Kum Hee Lee[2], Byoungki Choi[2], Seung-Yeon Kwak[2,*], Jun Yeob Lee[1,*]

[1]School of Chemical Engineering, Sungkyunkwan University

2066, Seobu-ro, Jangan-gu, Suwon, Gyeonggi-do, 16419, Republic of Korea

[2]Samsung Advanced Institute of Technology, Samsung Electronics

Suwon, Gyeonggi-do, 16678, Korea.

E-mail: leej17@skku.edu

* To whom correspondence should be addressed.

Synthesis of the materials

General

Unless otherwise noted, all commercially available chemicals were used without further purification after purchase from Sigma-Aldrich and Tokyo Chemical Industry (TCI). Phenylpyridine type ligands were purchased from Hanchem, Co., and used without further purification. NMR spectra recorded on Bruker ASCEND 500 (500MHz for 1H NMR, 125 MHz for 13C NMR). Chemical shift are given in ppm with the residual solvent signal as internal standard (dichloromethane at 5.32 ppm and 54.00 ppm, respectively). Mass spectrometry (MS) analysis was performed with LCMS-IT-TOF.

Materials

Standard synthesis procedure

$Ir(COD)_2BF_4$ (3.0 g, 6.06 mmol) and phenylpyridine ligand (3.3 equiv.) were dissolved in 100 mL of 2-ethoxyethanol, and stirred under 110 °C for 24 Hrs. After cooling down, yellow precipitates were filtered, and washed with excess amount of methanol to give crude product over 90% purity. The yellow powder was further purified with silica column chromatography (Eluent: Hex/EA), and recrystallized with DCM/MeOH to give the desired product over 99% purity.

Characterization of the products

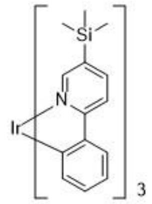

GD1: yellow powder (1.15 g, 22%); ^{1}H-NMR (500MHz, CH_2Cl_2) : δ (ppm) = 7.94 (d, *J* = 7.6Hz, 1H), 7.80 (dd, *J* = 8.03, 1.64Hz, 1H), 7.74 (m, 1H), 7.52(dd, *J* = 1.52, 0.84Hz, 1H), 6.95 (m, 2H), 6.88 (m, 1H), 0.12 (s, 9H). ^{13}C-NMR (125MHz, CH_2Cl_2): δ (ppm) = 166.73, 161.50, 150.37, 144.02, 141.13, 136.99, 133.23, 129.84, 124.25, 119.74, 118.28. MS(m/z):$[M+H]^+$ calculated for $C_{42}H_{48}IrN_3Si_3$, 872.2858; found, 872.2867.

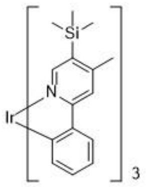

GD2: yellow powder (1.44 g, 26%); ^{1}H-NMR (500MHz, CH_2Cl_2) : δ (ppm) = 7.71 (s, 1H), 7.69 (m, 1H), 7.44(s, 1H), 6.91 (s, 2H), 6.84 (m, 1H), 2.55 (s, 3H), 0.13 (s, 9H). ^{13}C-NMR (125MHz, CH_2Cl_2): δ (ppm) = 166.51, 163.84, 153.09, 150.84, 145.42, 136.94, 131.65, 129.56, 124.01, 119.58, 119.50, 22.48, -1.01. MS(m/z):$[M+H]^+$ calculated for $C_{45}H_{54}IrN_3Si_3$, 914.3328; found, 914.3331.

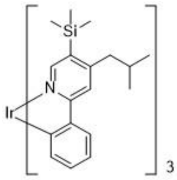

GD3: yellow powder (1.05 g, 17%); ^{1}H-NMR (500MHz, CH_2Cl_2) : δ (ppm) = 7.76 (s, 1H), 7.72 (d, *J* = 7.6Hz, 1H), 7.57(s, 1H), 6.92 (m, 1H), 6.86 (m, 2H), 2.69 (m, 2H), 2.13 (dt, *J* = 13.5, 6.8Hz, 1H), 1.04(t, *J* = 6.8Hz, 6H) 0.14 (s, 9H). ^{13}C-NMR (125MHz, CH_2Cl_2): δ (ppm) = 166.19, 162.07, 156.55, 151.61, 144.22, 136.94, 132.07, 129.6, 124.05, 119.44, 118.55, 44.66, 28.93, 22.32, 22.25, -0.01. MS(m/z):$[M+H]^+$ calculated for $C_{54}H_{72}IrN_3Si_3$, 1040.4736; found, 1040.4749.

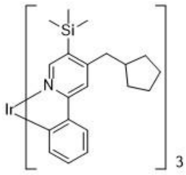

GD4: yellow powder (1.39 g, 21%); ^{1}H-NMR (500MHz, CH_2Cl_2) : δ (ppm) = 7.80 (s, 1H), 7.72 (dd, *J* = 7.8, 0.98Hz, 1H), 7.52(s, 1H), 6.89 (m, 3H), 2.83 (dd, *J* = 7.3, 4.51Hz, 2H), 2.26 (m, 1H), 1.88 (m, 2H), 1.72 (m, 2H), 1.61 (m, 2H), 1.30 (m, 2H), 0.13 (s, 9H). ^{13}C-NMR (125MHz, CH_2Cl_2): δ (ppm) = 166.26, 162.09, 157.21, 151.40, 144.24, 136.93, 131.70, 129.58, 124.04, 119.40, 118.29, 41.64, 40.55, 32.72, 24.95, 24.92, -0.15. MS(m/z):$[M+H]^+$ calculated for $C_{60}H_{78}IrN_3Si_3$, 1118.5206; found, 1118.5210.

Supplementary Figures

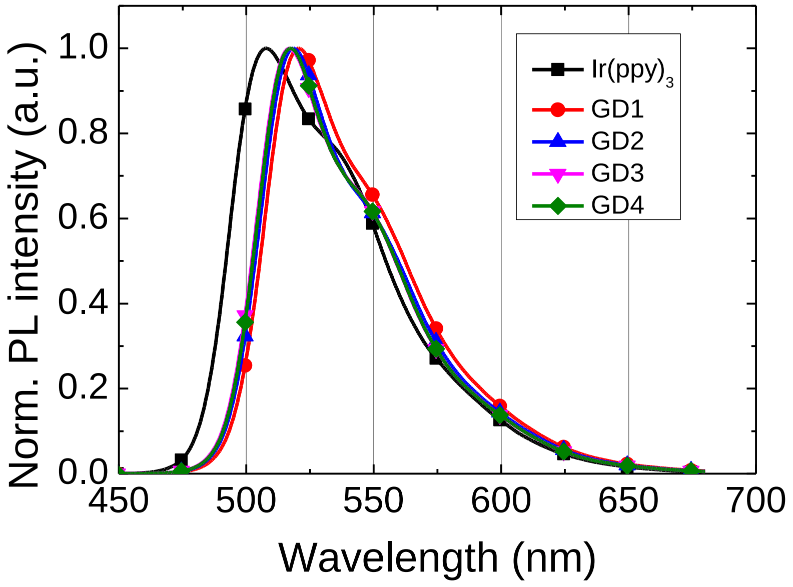

Figure S1. PL spectra of homoleptic Ir complexes in toluene solution. The concentration was 10^{-5} M.

	HOMO	LUMO
Ir(ppy)$_3$	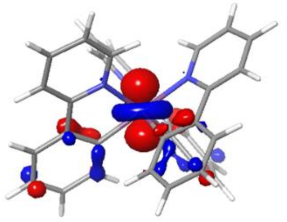	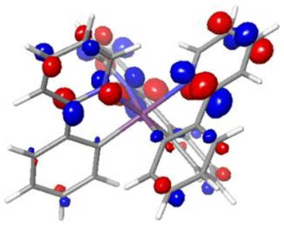
GD1	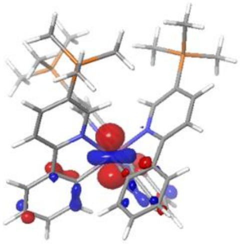	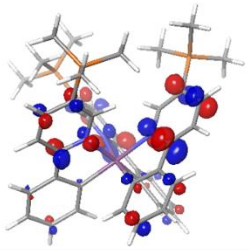
GD2	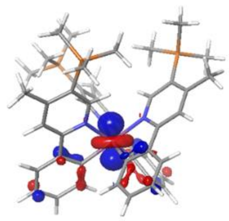	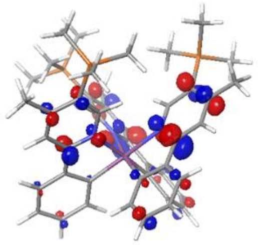
GD3	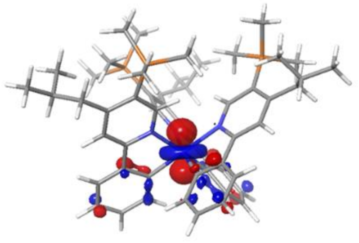	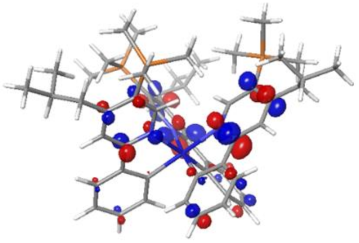
GD4	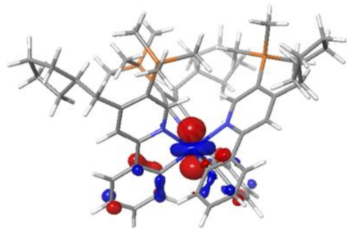	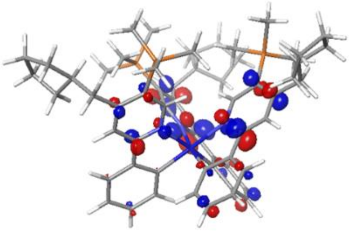

Figure S2. Distribution of HOMO and LUMO of homoleptic Ir complexes.

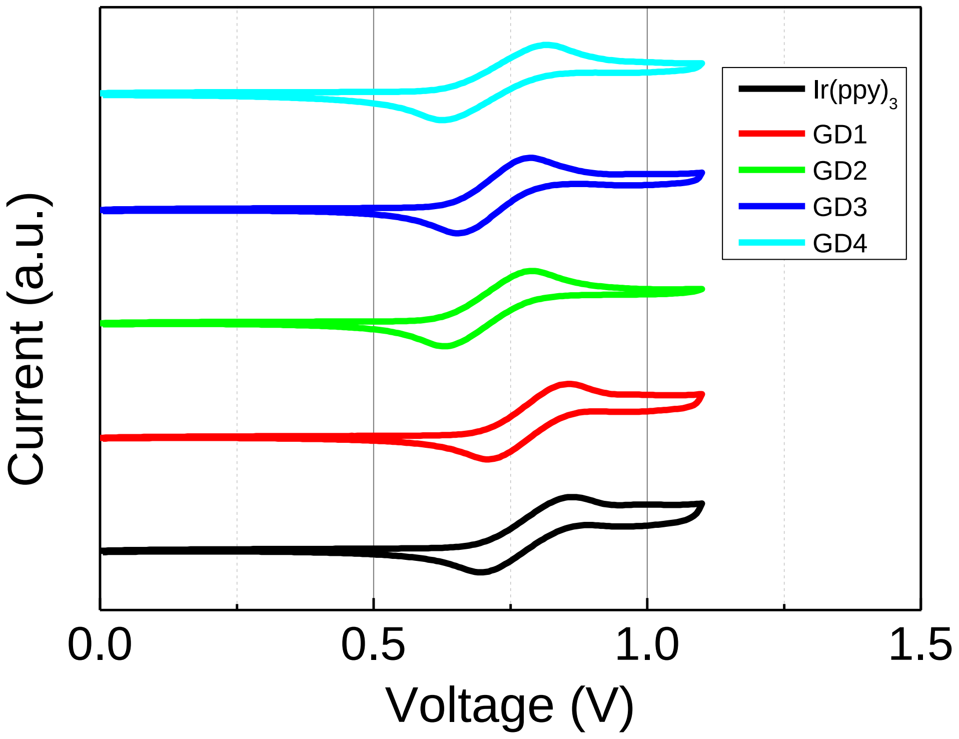

Figure S3. Cyclic voltammetry curves for the oxidation of green phosphorescent Ir complexes dissolved in methyl chloride with electrolyte.

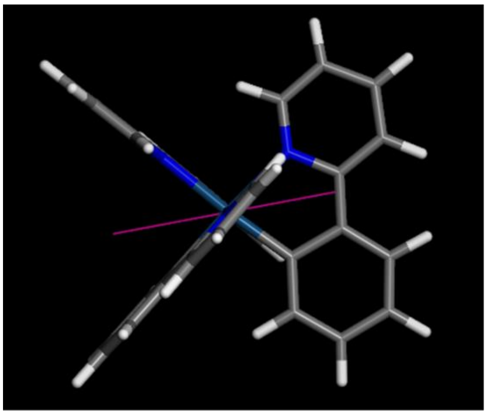

$Ir(ppy)_3$

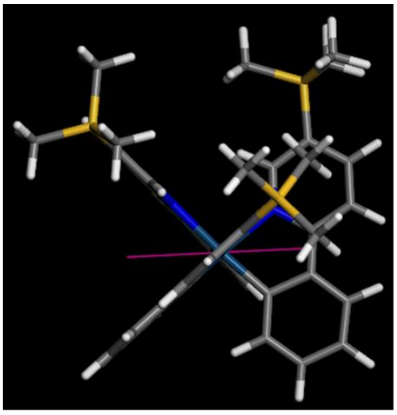

GD1

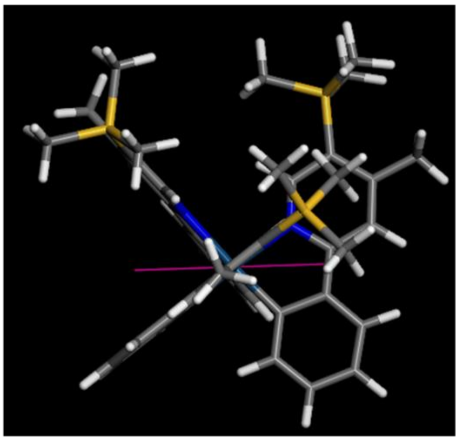

GD2

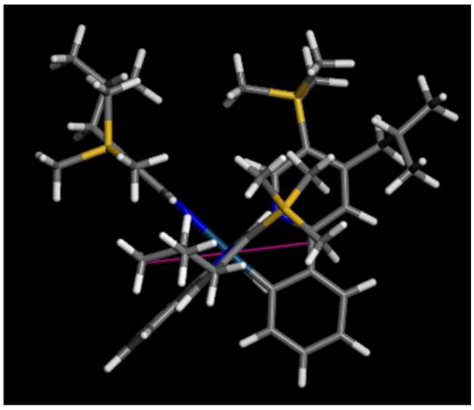

GD3

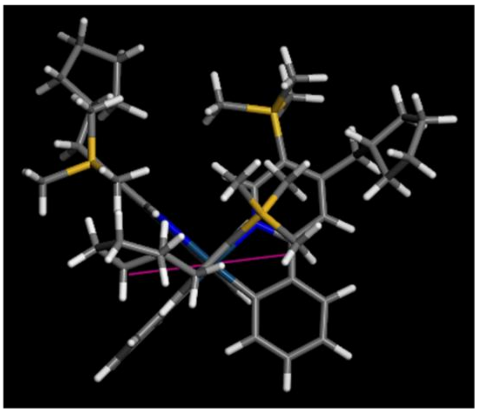

GD4

Figure S4. Orientation of the transition dipole moment vector with optimized geometry.

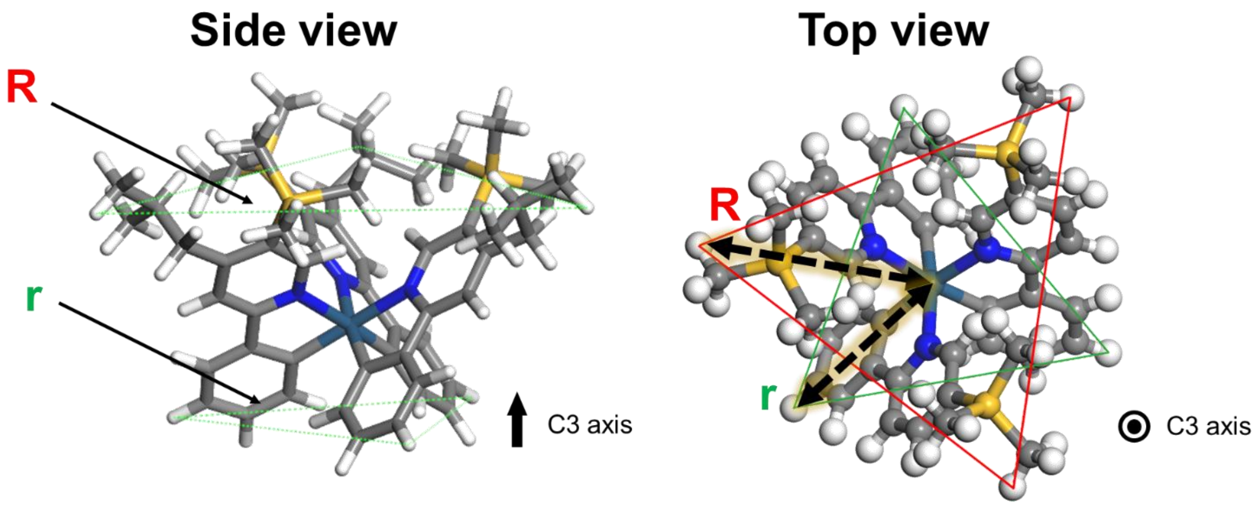

Figure S5. Schematic illustration describing the definition of the geometric anisotropic factor (aspect ratio) of the homoleptic Ir complexes in this work.

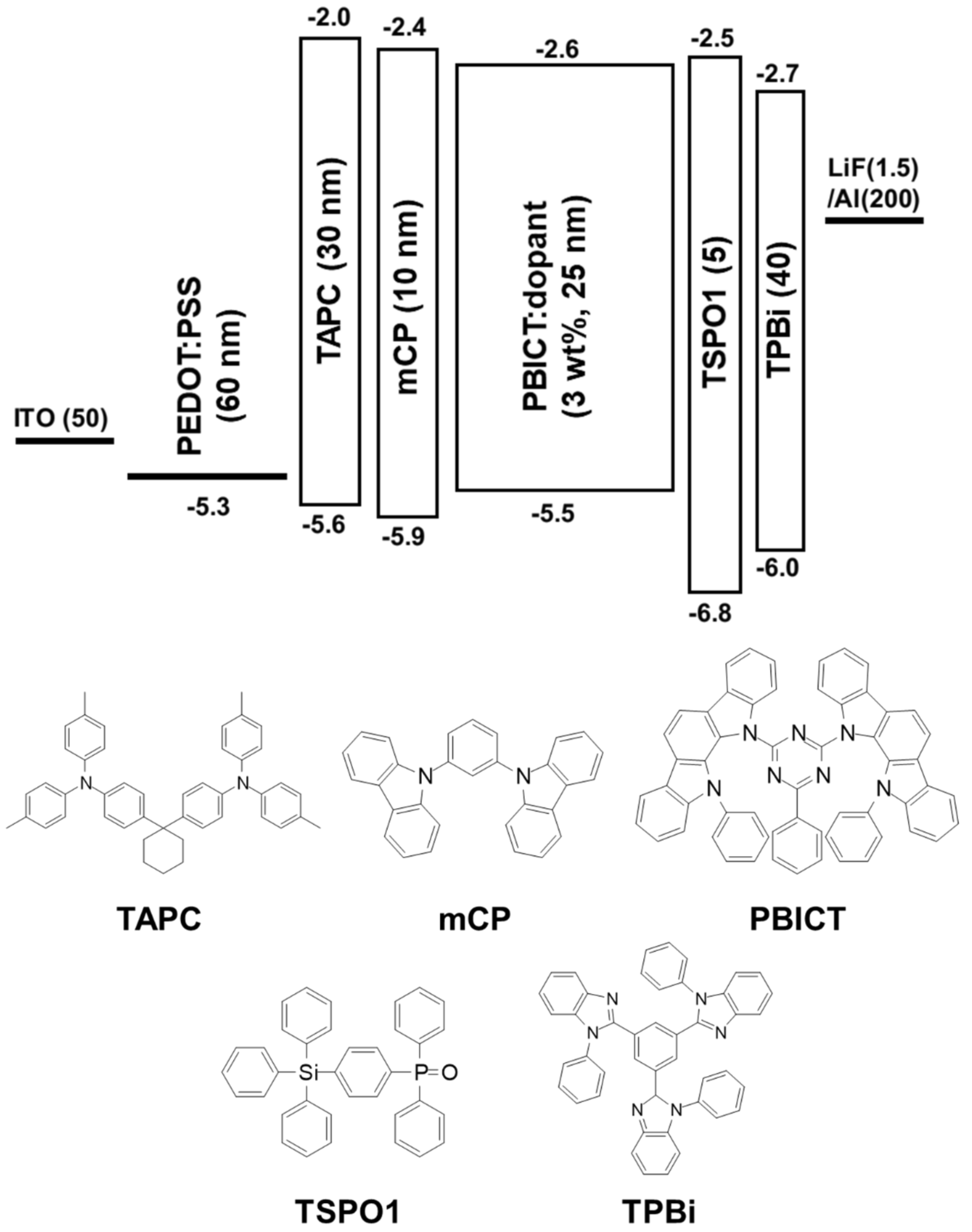

Figure S6. Device architecture and molecular structures of materials in PhOLEDs.

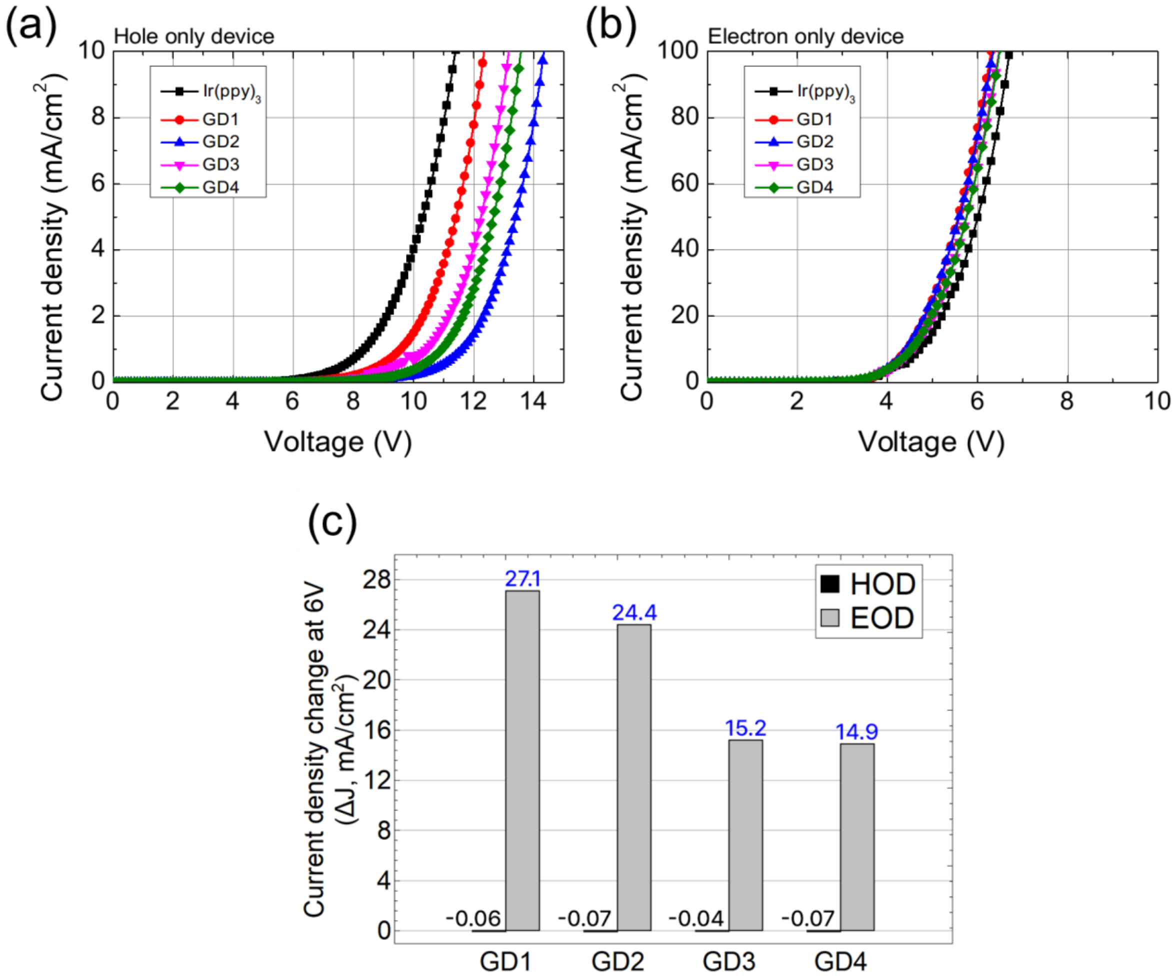

Figure S7. Current density-voltage characteristics of (a) hole-only device and (b) electron-only devices. (c) The difference of the current densities at 6V in the single charge devices between GD1~4 device and $Ir(ppy)_3$ device. The device structures of the single carrier devices were indium tin oxide (ITO)/PEDOT:PSS (60 nm)/1,1-bis[4-di(p-toluyl)aminophenyl]cyclohexane (TAPC, 10 nm)/1,3-Bis(N-carbazolyl)benzene (10 nm)/EML (25 nm)/TAPC (10 nm)/Al (200 nm) for the hole-only devices and ITO/2-[4-(9,10-di-naphthalen-2-yl-anthracen-2-yl)-phenyl]-1-phenyl-1H-benzoimidazole (10 nm)/EML (25 nm)/diphenylphosphineoxide-4-(triphenylsilyl)phenyl (5 nm)/2,2,2-(1,3,5-benzinetriyl)-tris(1-phenyl-1H-benzimidazole) (10 nm)/LiF (1.5 nm)/Al (200 nm) for the electron-only devices.

^{1}H and ^{13}C NMR spectra

GD1

^{1}H NMR

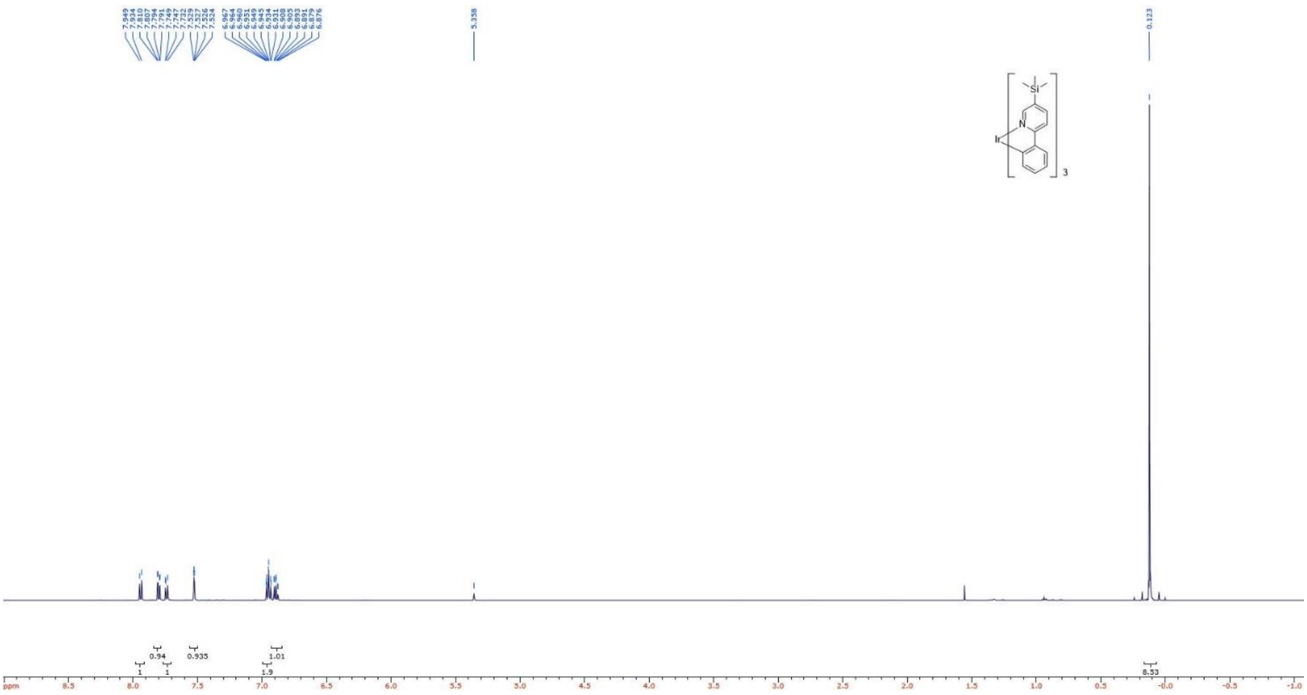

^{13}C NMR

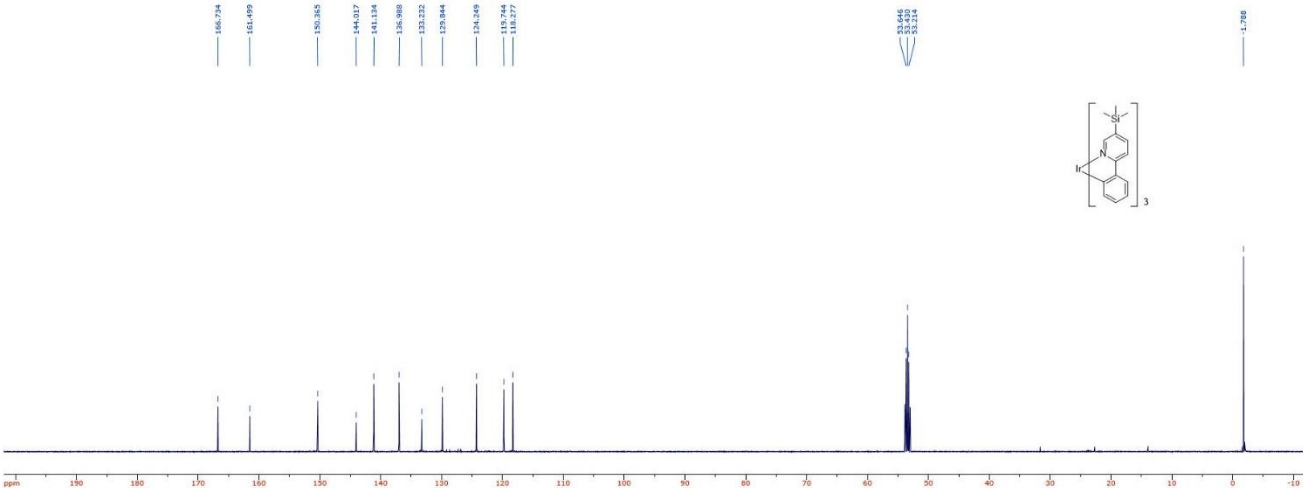

GD2

^{1}H NMR

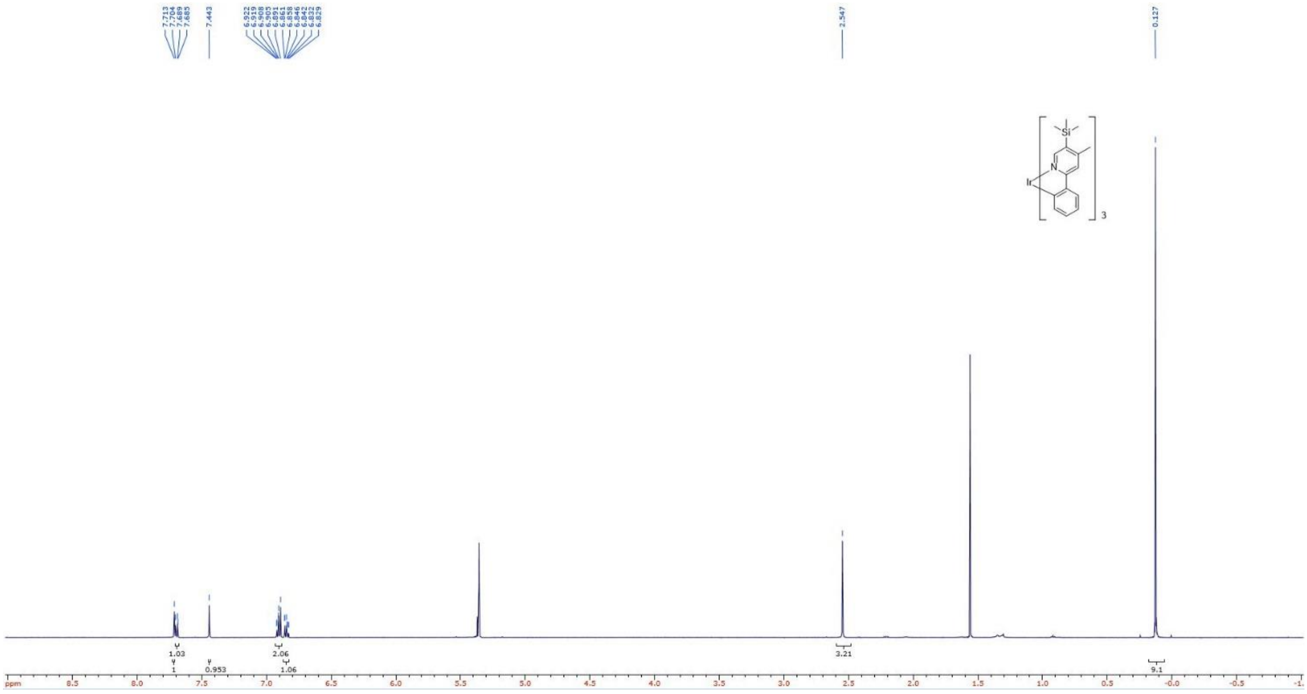

^{13}C NMR

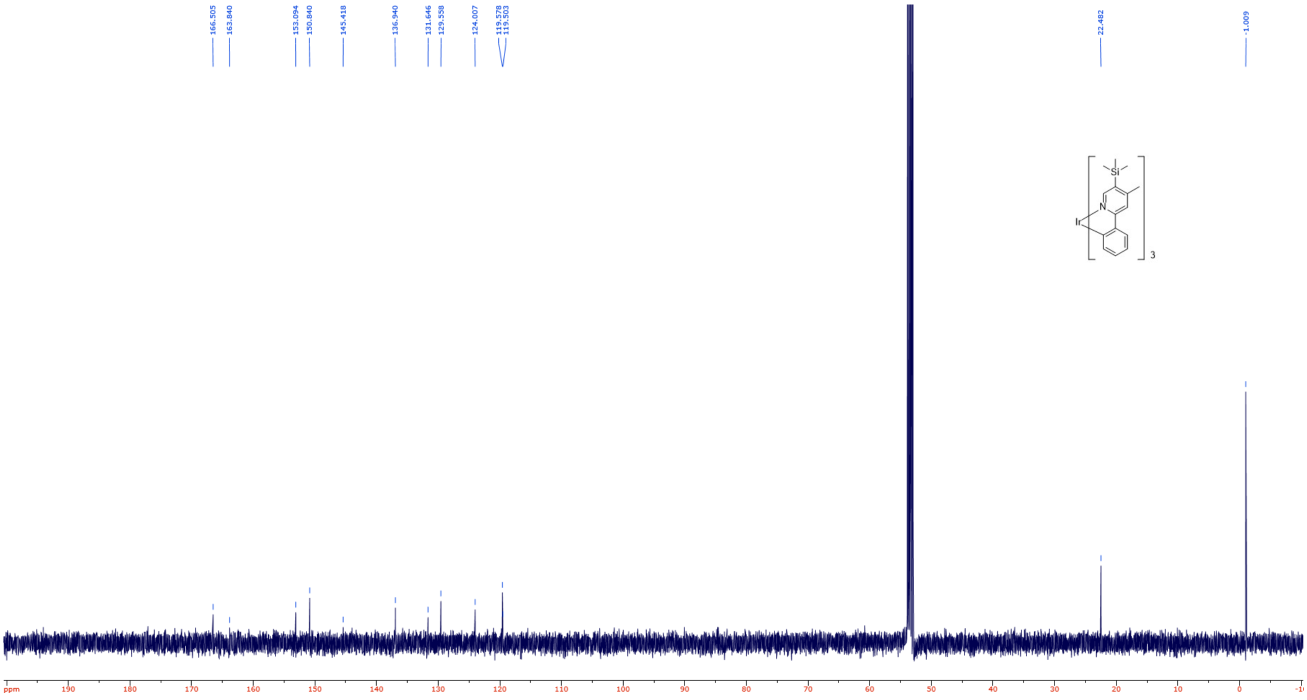

GD3

^1H NMR

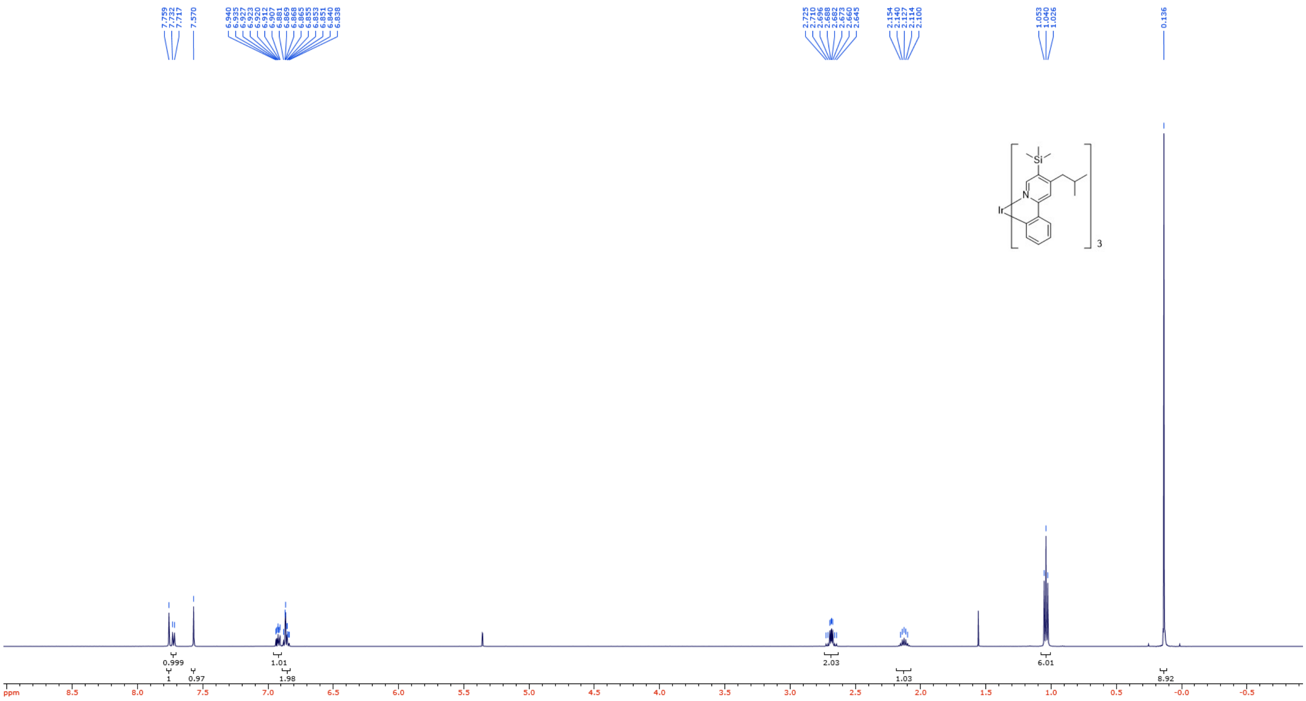

^{13}C NMR

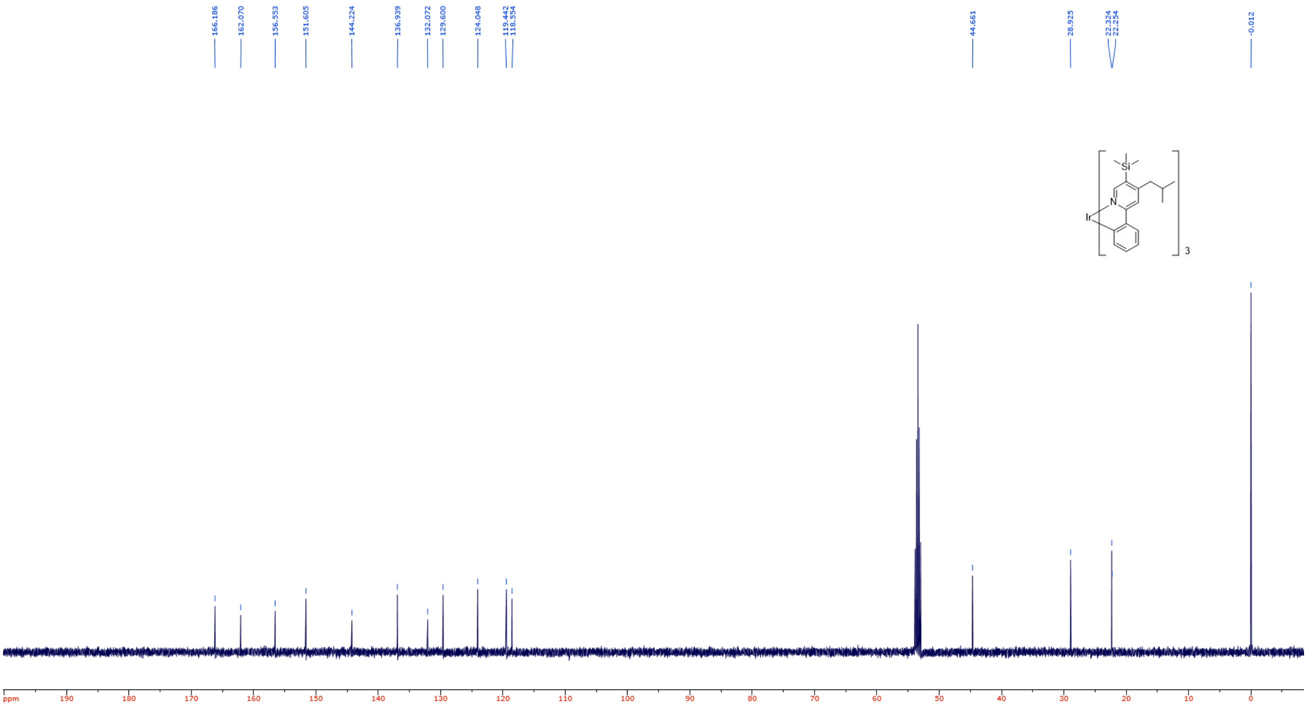

GD4

^{1}H NMR

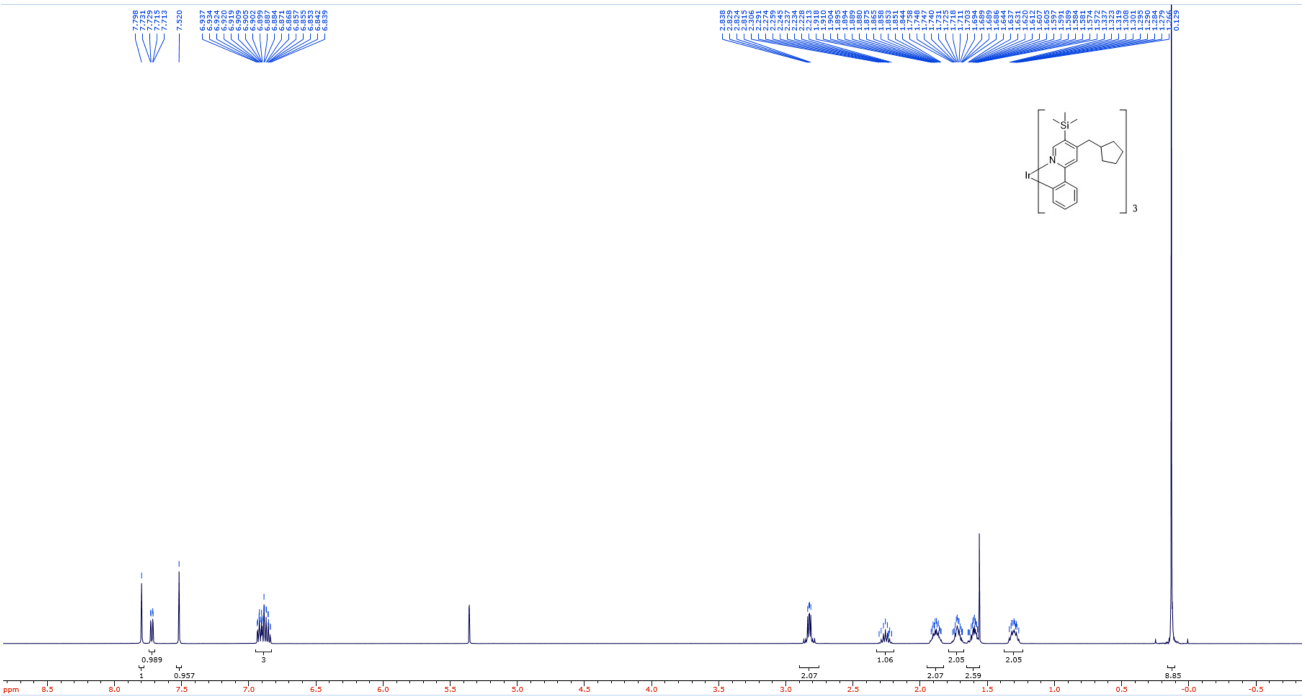

^{13}C NMR

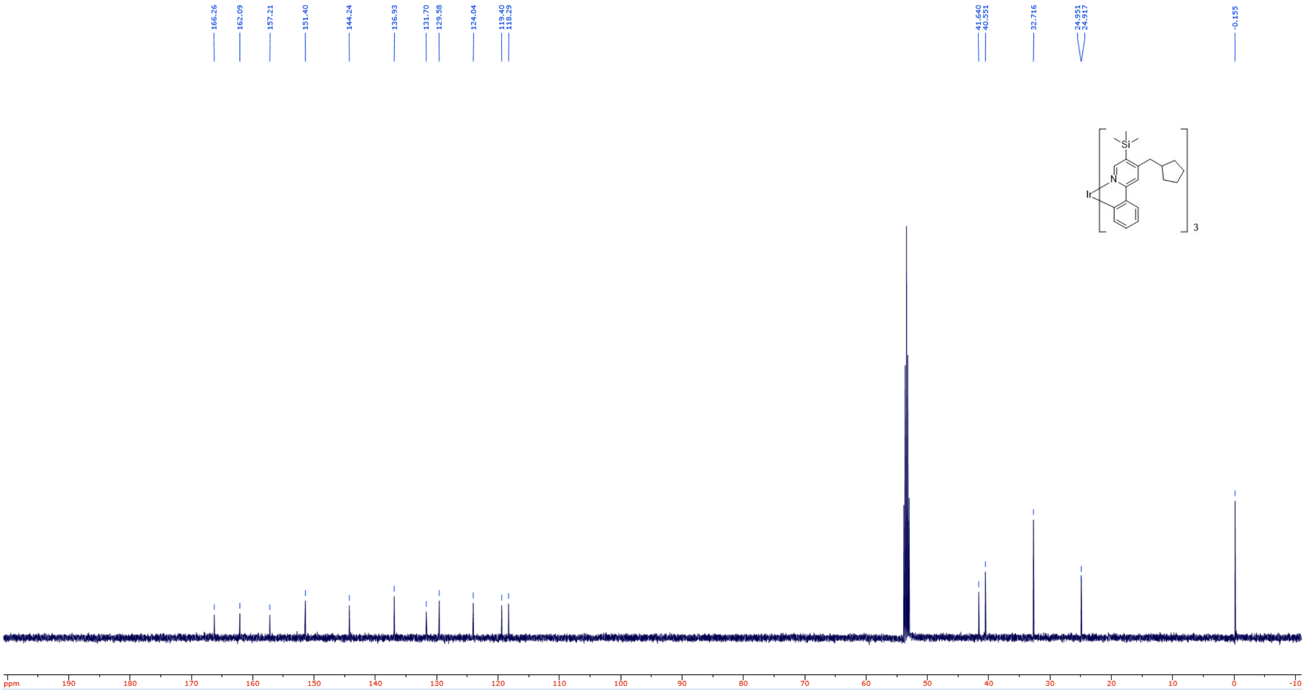

Supplement: Supplementary file 1 — Supporting Information [file ADVS-9-2203903-s001.pdf]
